# Co-Design and Non-Randomised Pilot Evaluation of Resources Developed to Optimise Saliva Management in People with Motor Neurone Disease

**DOI:** 10.3390/healthcare13212813

**Published:** 2025-11-05

**Authors:** Shana Taubert, Annette Collins, Robert Henderson, Pamela McCombe, Lily Tang, Katrina Kramer, Laurelie Wishart, Clare Burns

**Affiliations:** 1 The Royal Brisbane and Women’s Hospital, Brisbane, QLD 4029, Australia; annette.collins@health.qld.gov.au (A.C.); robert.henderson@health.qld.gov.au (R.H.); pamela.mccombe@health.qld.gov.au (P.M.); lily.tang@health.qld.gov.au (L.T.); katrina.kramer@health.qld.gov.au (K.K.); clare.burns@health.qld.gov.au (C.B.); 2 The University of Queensland Centre for Clinical Research, Brisbane, QLD 4029, Australia; 3 School of Medicine and Dentistry, Griffith University, Brisbane, QLD 4111, Australia; l.wishart@griffith.edu.au; 4 Office of the Chief Allied Health Practitioner, Metro North Health, Brisbane, QLD 4029, Australia; 5 School of Health & Rehabilitation Sciences, The University of Queensland, Brisbane, QLD 4072, Australia

**Keywords:** motor neurone disease, saliva, sialorrhoea, secretions, self-management, symptom management, multidisciplinary management

## Abstract

**Background/Objectives**: People living with MND (plwMND) commonly develop difficulty swallowing and subsequent difficulty clearing saliva from the airway. Medical saliva interventions include pharmacological agents, botulinum toxin injections, and radiation to salivary glands, with associated side effects. Non-invasive behavioural strategies and natural remedies are also recommended. Saliva symptom management is guided by the multidisciplinary MND team (typically through a three-monthly clinic) alongside community clinicians. Some plwMND report difficulty recalling and implementing treatments between clinics. This study aimed to enhance the content and method of providing recommendations for self-management of saliva symptoms by (i) developing MND-specific resources and (ii) evaluating resource use and preliminary clinical benefit. **Methods**: In Phase 1 plwMND, caregivers, and clinicians co-designed saliva management resources. Phase 2 examined the use of these resources via a hospital-based MND clinic with 28 plwMND, their caregivers, and community clinicians. In the clinic, plwMND were given a written treatment plan and relevant resources. During reviews at weeks 2, 6, and 12 saliva treatment was adjusted and clinical outcomes evaluated using the Clinical Saliva Scale for MND (CSS-MND). Community clinicians, plwMND, and caregivers were surveyed regarding the resource utility. **Results**: People living with MND reported the resources assisted saliva symptom self-management. Community clinicians found the resources informative and beneficial in supporting patient care. All plwMND required multiple treatment strategies and adjustments to manage symptoms. Of the treatments prescribed, 91% were non-invasive and 9% were medical interventions. For 54% (*n* = 15) of plwMND, improved CSS-MND scores were sustained over the three-month evaluation. **Conclusions**: Co-designed saliva resources and regular reviews assisted plwMND to implement their individualised saliva treatment, to self-manage saliva symptoms between clinics.

## 1. Introduction

Motor Neurone Disease (MND) is a neurodegenerative disorder causing progressive loss of muscle function to a variable degree across limb, bulbar, and respiratory muscles, resulting in significant disability [1]. Many people living with MND (plwMND) will develop a swallow impairment as the disease progresses, with almost half of plwMND experiencing difficulty swallowing saliva [2]. Due to orofacial muscle weakness, unswallowed saliva spills from the mouth, hinders speech, and causes social distress [3]. Unswallowed saliva may accumulate in the pharynx/throat, with over 70% of plwMND demonstrating inefficient pharyngeal clearance early in the disease trajectory [4]. Concomitant ineffective cough prevents saliva expulsion from the upper airway, causing the sensation of choking and difficulty breathing [5,6]. Further, accumulated saliva increases the risk of aspiration pneumonia and hinders the use of non-invasive ventilation, which impacts survival [5,7]. As MND remains incurable, clinical care aims to manage symptoms, limit distress, and optimise quality of life [8].

Saliva management for plwMND is complex due to heterogeneity among MND clinical subtypes and limited evidence regarding the effectiveness of saliva therapies [2,9,10,11]. Medical treatments include anticholinergic medications, botulinum toxin injections, and radiation to salivary glands, with side effects including dry mouth, constipation, and delirium [9,11,12]. Non-invasive approaches, including behavioural strategies (e.g., hydration and steaming) and natural remedies (e.g., papaya enzyme), are recommended by some MND specialists, but lack published evidence [11,13,14,15]. Specific saliva characteristics and causative factors require different treatments, informed by accurate and comprehensive assessment, with regular review to identify side effects, ineffective therapy, or disease progression necessitating treatment adjustment [2,15,16,17,18].

Our facility operates a specialist MND clinic offering approximately three-monthly reviews jointly by a multidisciplinary team incorporating neurologists, palliative medicine specialists, a specialist nurse, a dietitian, and a speech pathologist (SLP), with interim follow-up community-based care. During MND clinic visits, information and advice are provided regarding saliva management, among other topics. Providing unfamiliar information under stressful conditions may hinder recall [19]. Often, plwMND and their caregivers tell us that clinics feel overwhelming, with subsequent difficulty remembering and implementing their recommended saliva management strategies. Even when managed by MND specialists, saliva symptoms remain uncontrolled in almost half of plwMND presenting with saliva problems [11,20], with resulting symptom escalation and distress.

Therefore, this study aimed to enhance the content and method of providing recommendations for self-management of saliva symptoms between three-monthly MND clinics by (i) developing MND-specific saliva management multimedia resources and (ii) examining resource use and preliminary clinical benefit over a 3-month period. The aim of these resources was to support plwMND, caregivers, and healthcare professionals in clinical decision-making to support self-management of saliva symptoms, at home between MND clinic appointments.

## 2. Materials and Methods

This mixed-methods study was conducted in two phases. Phase 1 involved the co-design of a saliva decision-making framework with the MND multidisciplinary team, alongside instructional consumer resources co-designed by the MND team, plwMND, and their caregivers. Phase 2 involved a non-randomised pilot evaluation of the saliva decision-making framework and resources with a cohort of plwMND and subsequent examination of the utility of the resources and preliminary clinical outcomes.

A waiver of ethical consent was granted by the Hospital and Health Service Human Research Ethics Committee to conduct this study.

### 2.1. Study Setting

The study was conducted at the MND clinic of an Australian quaternary public hospital. This clinic offers three-monthly appointments to plwMND residing within a large geographical area, resulting in ~300 appointments annually. The multidisciplinary team provides joint consultations during MND clinic appointments.

### 2.2. Phase 1—Co-Design of Saliva Decision-Making Framework and Resources

**Figure 1:**
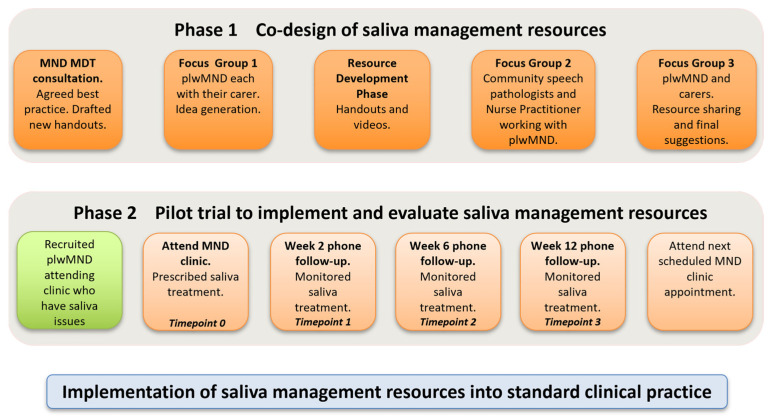
Overview of study phases.

The saliva decision-making framework and resources were developed iteratively (see Figure 1). Initially, MND team members and pharmacists discussed and agreed on best practice methods for saliva management in MND based on current evidence and expert knowledge. Based on these findings, a decision-making framework for MND clinicians to identify and prescribe appropriate treatments for plwMND was confirmed. The SLPs then drafted instructional consumer resources regarding managing saliva (thick and thin) and dry mouth, performing mouth care and humidification, using natural remedies, as well as a saliva monitoring chart for plwMND to document their saliva symptoms across the day for three days.

Next, plwMND attending the clinic (who were experiencing saliva problems) were informed of the study. Three plwMND and caregiver dyads participated in two focus groups led by the SLPs (ST, AC). The first focus group sought to understand participant’ experiences of saliva issues and elicit their ideas to further inform content for instructional resources. The focus group reconvened to review and endorse the final versions, and participants also contributed to short videos to supplement the written resources. They provided written informed consent to do so.

Community clinicians providing services to people attending the MND clinic were emailed invitations to attend a single focus group via videoconference. Eight SLPs and one palliative care nurse practitioner attended. This meeting sought to understand their information requirements to support plwMND with saliva management and also to seek their feedback on the saliva decision-making framework and the consumer resources developed. Written notes and audio recordings made during all group meetings were used to inform the development and revision of the resources. All stakeholder meetings were conducted in accordance with the health service’s consumer and community engagement guidelines.

### 2.3. Phase 2—Pilot Evaluation of the Saliva Decision-Making Framework and Resources

A non-randomised pilot evaluation was conducted with a cohort of plwMND. From July to November 2023, all plwMND attending the MND clinic who reported saliva issues were advised of the study and invited to participate (+/− their caregivers). PlwMND with cognitive and communication issues were not excluded, provided that they could communicate via assisted or alternative communication and/or had appropriate caregiver support to participate. 

**Figure 2:**
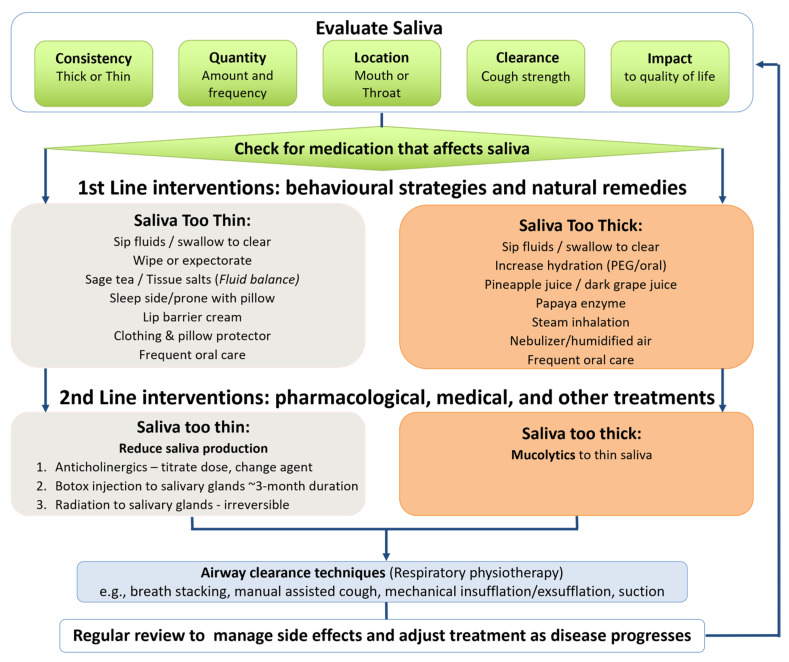
MND decision-making framework for saliva management.

At the clinic appointment, the MND team used the decision-making framework (see Figure 2) to prescribe individualised saliva treatment and provide instructional resources. Immediately after their MND clinic appointment, those plwMND who agreed to participate in the study +/− their caregiver, met separately with the research SLP (AC), who was not present in the clinic. The researcher collected baseline data regarding saliva status, checked participants’ understanding of the saliva treatment prescription, discussed its implementation at home, and provided access to the online instructional consumer resources. Each participant was asked to complete the saliva monitoring chart prior to review appointments. The participant’s community SLP received a copy of the saliva treatment prescription and links to the recommended consumer resources, as part of the usual clinical handover.

Participants then attended three review appointments at weeks 2, 6, and 12 following the clinic appointment. At these appointments, the research SLP (AC) repeated saliva assessments, recorded current saliva issues, discussed whether the recommended prescription was implemented, undertook problem-solving, and then adjusted the saliva treatment prescription, guided by the saliva decision-making framework. Data on the use of the resources was also collected. Following the 12-week review appointment, all plwMND +/− caregivers and their treating community SLPs provided survey feedback on their satisfaction with the saliva resources.

### 2.4. Data Collection

Demographic data was collected relating to: (a) characteristics of plwMND: sex, age, location of residence; (b) MND characteristics; (c) caregiver characteristics; and (d) details of community SLP. Clinical data recorded at each time point (i.e., Time point 0 = MND clinic, Time points 1–3 = review appointments) included:

(a) The Clinical Saliva Scale for MND (CSS-MND): A validated patient-reported outcome measure evaluating the severity and impact of saliva issues. The scale comprised 10 questions, each with a 4-point scale (0 = no issues or symptoms; 3 = significant issues) evaluating saliva consistency, severity, frequency, location, and impact on daily activities. We incorporated the two additional questions, suggested by the Scale’s authors, in their Supplementary Materials [2].
(b) Two survey questions regarding the overall impact of saliva changes on daily life, and frequency of experiencing dry mouth symptoms, both rated on a 5-point Likert scale, developed by our research team (0 = never; 4 = very often; see Supplementary Materials).
(c) Patient-reported saliva symptoms.
(d) Adherence to prescribed saliva treatment and issues associated with implementing recommendations.
(e) Adverse events associated with saliva issues.
(f) Changes to saliva prescription.

Following the three-month pilot evaluation, plwMND, caregivers, and community SLPs were emailed a link to an online survey seeking feedback on the saliva resources (see Supplementary Materials). Finally, service data recorded included appointment attendance, duration, and modality (telephone, telehealth, email, in person).

### 2.5. Data Analysis

Descriptive statistics were utilised to analyse participant demographics, service data, and consumer use and feedback regarding the saliva resources. Statistical analysis of the intervention regarding overall treatment outcome (CSS-MND Total score) over time was explored in two ways. First, univariable analysis (ordinary least squares regression) was conducted to explore crude associations between the assessment time point and the outcome. Multivariable modelling, using mixed-effects general linear modelling (GLM) with Gaussian assumptions, was then used to adjust for potential confounding of individual participant variability, and known impacts of presenting saliva type and MND onset type. For the GLM analysis, assessment time point, MND onset type (limb/bulbar) and presenting saliva type (thin/thick) were forced into the model as fixed effects, and participant ID was entered as a random effect to control for individual variability. This mixed-effects modelling is appropriate for repeated-measures data, particularly when sample sizes are modest, and includes missing or correlated data [21]. One observation from one participant (p13, Time point 2) was removed for this analysis as it was a significant outlier from the cohort, and another participant was excluded (p10) as their saliva type could not be dichotomised. Model fit was checked using the Akaike Information Criterion (AIC). Normality of the multivariable model was assessed using histograms and patterns of variance using rvf (residual vs. fitted values) plots. Results were analysed using Stata 19. For all results, statistical significance was set at *p* < 0.05, with 0.05 > *p* > 0.07 discussed as trends.

## 3. Results

### 3.1. Phase 1—Co-Designed MND Saliva Decision-Making Framework and Resources

The three stakeholder groups co-designed a range of saliva management resources. These incorporated the following:

(a) Saliva monitoring chart: Completed by plwMND to document saliva symptoms and additional coughing/distress at various time points across three days to inform saliva management prescription and required adjustments by MND clinicians.
(b) Saliva decision-making framework (see Figure 2): Guides MND clinicians in saliva evaluation and then provides a hierarchy of interventions to trial. The framework prioritises non-invasive behavioural strategies/natural remedies as first-line interventions and pharmacological/medical treatments as second-line interventions. A simplified version of the framework was created for plwMND, with directions to complete the saliva monitoring chart to inform treatment strategies and contact information for additional support.
(c) Saliva Treatment Prescription Template: Supports standardised documentation and clear communication of prescribed saliva management strategies among the MND Clinic team, plwMND, caregivers, and community health professionals.
(d) Consumer Saliva Management Resources: A suite of consumer materials including handouts and instructional videos addressing thin saliva, thick saliva, dry mouth, mouth care, natural remedies, and steam inhalation. Each handout includes a QR code linking to a video demonstration of the strategies to enable management at home. These resources are publicly available via the Metro North Health website (https://metronorth.health.qld.gov.au/rbwh/healthcare-services/neurology/motor-neurone-disease, accessed on 30 June 2025).

### 3.2. Phase 2—Pilot Evaluation Participants

Twenty-eight plwMND (mean age 62 years, range 38–87 years) participated in the pilot evaluation (See Table 1). Two-thirds were male, 50% had bulbar and 50% had limb onset types of MND, and >75% were >12 months post diagnosis. Half of the participants lived in metropolitan locations, 12 (43%) lived in regional locations, and 2 (7%) lived in rural locations. Except for the two rural participants, all received current community SLP support. Over 50% had a partner/family member as the caregiver participating in the evaluation.

### 3.3. Attendance at Follow-Up Appointments

Of the 28 participants who attended the baseline appointment, 15 attended three further scheduled reviews, 10 only attended two reviews, and 3 only attended one review. Reasons for non-attendance were health deterioration (*n* = 1), death (*n* = 1), extended holiday (*n* = 1), and nursing home staff unavailability to facilitate the appointment (*n* = 2). Thirteen plwMND did not complete all scheduled appointments as the evaluation period ended before these time points could be reached, due to significant administrative delays with study commencement. Baseline assessment coincided with MND clinic appointments, occurring in-person (*n* = 27) or via videoconference (*n* = 1). Review appointments occurred via phone (*n* = 56), videoconference (*n* = 1), or email (*n* = 5) due to speech difficulties, or in person (*n* = 3). The mean appointment duration was 22 min.

### 3.4. Characteristics of Saliva and Saliva-Related Symptoms

Saliva characteristics derived from the CSS-MND and participant-reported symptoms are displayed in Table 1. Sixty-one percent of participants (*n* = 17) had predominantly thin saliva, while 36% (*n* = 10) had predominantly thick saliva. Over 90% (*n* = 25) experienced saliva pooling in both the mouth and throat. Dry mouth was reported by 42% (*n* = 12), co-existing with both thin saliva and thick saliva. Secretion symptoms reported in addition to (or instead of) pooled saliva included reflux (*n* = 3), postnasal drip (*n* = 2), or candidiasis of the mouth or throat (*n* = 2).

### 3.5. Treatments Prescribed for Saliva and Saliva-Related Symptoms

Various treatments were prescribed, specifically targeting thin saliva or thick saliva characteristics. Most plwMND required more than one treatment prescribed at the initial appointment, with additional or alternative treatments, or dose adjustments, required at subsequent appointments. Across all four time points, half of the participants (*n* = 14) had 4–5 different treatments prescribed, 29% (*n* = 8) had 2–3 treatments prescribed, and 21% (*n* = 6) had 6–10 treatments prescribed.

Across all time points, 51% of all treatments prescribed were behavioural and 38% were natural remedies (Table 2). Eleven percent were second-line interventions prescribed by physicians. Pharmacological agents prescribed were glycopyrrolate and amitriptyline. Botulinum toxin and radiation were prescribed each on one occasion. Regarding non-saliva secretions, three treatments were prescribed for postnasal drip, three for candidiasis, and four for reflux. No adverse events occurred relating to saliva symptoms or treatments during the study.

At the first follow-up appointment, 79% (*n* = 22) of plwMND had implemented their prescribed saliva management strategies. This improved to 100% at the subsequent follow-up, with all plwMND requiring further detailed instruction or joint problem solving, which generated simple solutions, to optimise treatment implementation. Adjustments addressed dose, timing, or administration method of natural remedies, humidification, or simply increased oral/gastrostomy hydration. Helpful practical changes included setting up drinks to be independently accessible for those with reduced limb function, or small-sized servings of drinks to encourage frequent intake and reduce fear related to dysphagia.

### 3.6. Participant Reported Changes in Saliva and Saliva-Related Symptoms Following Treatment

Some improvements in saliva symptoms were reported by plwMND over the evaluation period, as indicated by a reduction in the CSS-MND total score. Descriptively, there was substantial individual variability in the trajectory of clinical presentation. Over half (54%, *n* = 15) of participants reported some degree of sustained improvement in saliva symptoms on the CSS-MND over the three months, while 25% (*n* = 7) maintained stable CSS-MND scores with no quantifiable therapeutic benefit. The remaining participants (11%; *n* = 3) reported worsening saliva symptoms or a mix of improvement and worsening across the evaluation period.

Statistical analysis conducted at the univariable level showed no significant association between assessment time point and CSS-MND total score. However, after adjusting for saliva type, MND onset type, and individual variability in the multivariable mixed-effects model, significant improvements in CSS-MND scores were observed in this participant cohort over time (Table 3). Specifically, there were significant reductions in scores from baseline to Time point 1 (*p* = 0.025) and baseline to Time point 2 (*p* < 0.0001), and a trend towards significance from baseline to Time point 3 (*p* = 0.070). The model also demonstrated that participants with bulbar onset MND had significantly worse overall saliva symptom scores than those with limb onset (*p* = 0.023). Regarding saliva type, there was no significant overall difference in symptom change between those with thin versus thick saliva.

### 3.7. Utilisation of and Satisfaction with Saliva Management Resources

**Figure 3:**
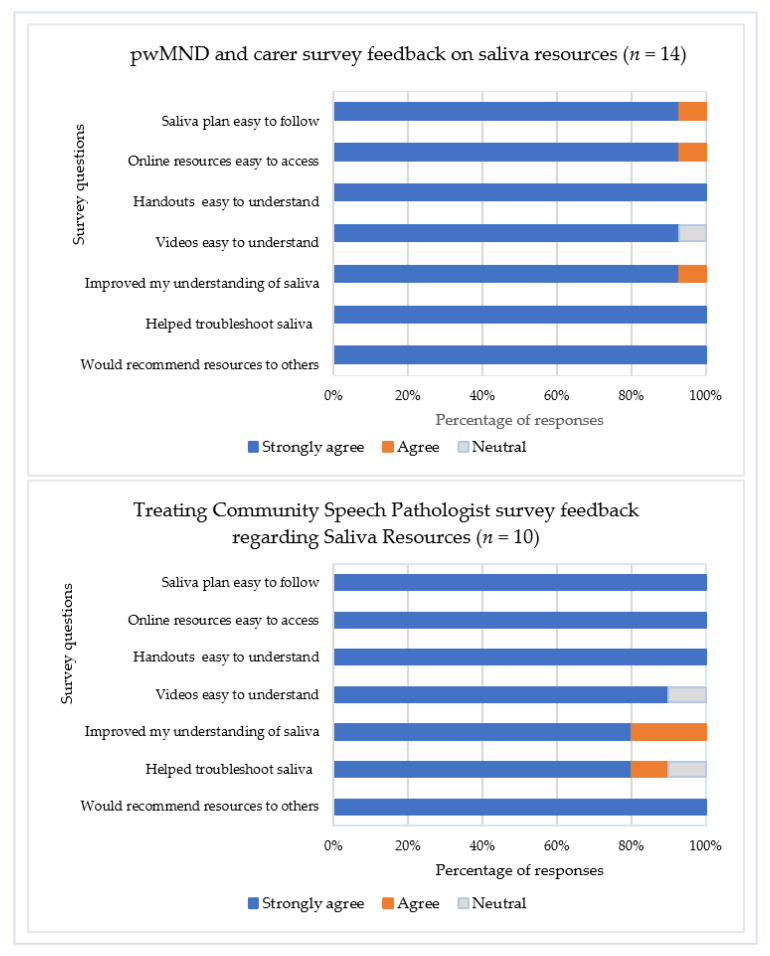
Survey responses regarding saliva resources from treating community speech pathologists, plwMND, and caregivers. *Note*. No participants responded ‘Disagree’ or ‘Strongly disagree’; plwMND = people living with MND.

All participants reported using the provided printed resources specific to their type of saliva problem, with most accessing online instructional videos. Survey feedback from community SLPs (*n* = 10) and plwMND/caregiver dyads (*n* = 14) confirmed high satisfaction with saliva management resources (Figure 3), with strong agreement that the saliva treatment plan, saliva management strategies, and instructional videos were easy to understand and apply to aid symptom management in the community. However, completion of the saliva monitoring charts was inconsistent, with less than 30% of plwMND (*n* = 8) completing the chart at Time point 1, 60% (*n* = 15) at Time point 2, and 50% (*n* = 8) of the plwMND who attended Time point 3. Consequently, participants suggested that an electronic version of the chart may be more convenient and less burdensome to complete, with an incoming email/text link serving as a reminder.

## 4. Discussion

In this study, an MND saliva decision-making framework was developed, and instructional resources were co-designed with plwMND, with the intention of supporting self-management of saliva symptoms. A three-month pilot evaluation of the framework and resources with a small non-randomised cohort of plwMND revealed high user satisfaction and positive preliminary outcomes regarding saliva management.

### 4.1. Treatments Prescribed for Saliva and Saliva-Related Symptoms

Our saliva decision-making framework guided the MND team’s standardised hierarchical treatment approach, beginning with non-invasive interventions. Consequently, ~90% of treatments prescribed were behavioural strategies and natural remedies. Often, we made simple treatment adjustments and prescribed multiple treatments addressing multifactorial problems, avoiding escalation to pharmacological or medical treatments. This approach differs from published findings reporting that a small proportion of clinicians used non-invasive treatments as first-line saliva interventions for plwMND [11]. Non-invasive treatments are typically recommended for thick secretions [13,14], and they often adequately control mild saliva symptoms in early-stage MND [17,18,20]. Moreover, they have fewer and easily reversible side effects, and they can be recommended by any MND health professional.

Second-line medical interventions require a prescribing professional—in our case, neurologists, palliative medicine specialists, or general practitioners. Four plwMND in our study were prescribed glycopyrrolate, and they all required at least one dose adjustment to address overthickening of saliva (which hinders effective clearance [22]) or ineffective treatment. Pharmacological agents commonly require titration, combination with other agents, or swapping to alternative anticholinergic agents to relieve salivary symptoms in MND [18,20], likely due to multifactorial causation of symptoms and disease progression. These dose titrations require consultation with prescribing practitioners, whereas non-invasive interventions can be adjusted by community clinicians during routine appointments.

### 4.2. Participant Reported Changes in Saliva and Saliva-Related Symptoms

For this pilot study, whole cohort results indicated promising preliminary outcomes following treatment supported by the saliva management resources. Our finding that 54% of plwMND showed saliva improvements on CSS-MND scores after treatment, which were maintained across time points, is positive in the context of a progressive disease. Earlier observational studies reported similar improvement rates (50–60% of plwMND had improved saliva symptoms after treatment) [11,20]. We found that individuals with bulbar onset MND had higher (worse) overall CSS-MND saliva scores than those with limb onset, consistent with prior studies [23], which likely reflects more severely impaired oropharyngeal swallow function with bulbar involvement [4]. Individual responses to treatments and the trajectory of saliva symptoms observed in our study were mixed, indicative of the complexity and variability of the MND-associated saliva symptoms observed in this study and in earlier research [11,18,20]. Further research with larger cohorts is needed to delineate the effects of clinical differences in disease stage, onset type, and saliva type.

### 4.3. Complexity and Variability of Saliva and Saliva-Related Symptoms

Consistent with the published literature, most plwMND in our study experienced variable combinations of saliva symptoms (i.e., thin/thick saliva, accumulation in the mouth/throat, dry mouth) [11,20]. This variability is determined by involvement of physiological factors, which may include (a) saliva spillage from the mouth due to lip and/or jaw weakness, (b) oral or pharyngeal pooling caused by weak swallowing [4], and (c) ineffective cough, preventing saliva clearance from the throat [6,10]. The seemingly paradoxical co-occurrence of thin saliva with mouth/throat dryness is associated with mouth breathing, non-invasive ventilation use, or inadequate fluid intake [23,24], and both thin and thick saliva can co-exist in over a third of plwMND [20]. In our study, saliva treatments for plwMND were tailored to account for saliva consistency (thin/thick), location (mouth/throat), and time of day [23]. Additionally, co-occurring reflux, postnasal drip, or candidiasis exacerbated saliva symptoms for some plwMND, requiring targeted questioning for accurate detection and separate treatment [25].

### 4.4. Resources

Using the saliva decision-making framework combined with the validated saliva assessment tool (CSS-MND) enabled prescription of targeted treatments and associated resources. This personalised service provision aimed to engage plwMND and caregivers in self-management, which is an important facet of MND care [26]. The decision framework guided clinicians to commence with non-invasive treatments, and supportive resources described how to apply these. Research speech pathologists led saliva reviews between clinics, escalating to the MND team when second-line treatments required consideration. Following this study, community clinicians who provide care to plwMND would be well placed to review saliva during their routine appointments. Using the structured decision framework and the evidence-based resources to support their decision-making may enhance community clinicians’ confidence in managing complex saliva issues in plwMND [27]. Sharing treatment plans and resources between clinic and community teams may improve communication and consistency of care to optimise saliva management for plwMND [28].

The high satisfaction and utilisation of the resources by plwMND confirmed their relevance and likely reflected end-users’ involvement in the co-design process [19]. Having videos and handouts online allowed plwMND to access information in their preferred format and facilitated revisiting the information and sharing with caregivers [19].

### 4.5. Monitoring

Notwithstanding the informative value of the resources, they are intended to supplement (not replace) regular monitoring by clinicians to support self-management. As MND progresses, review of saliva symptoms is critical to adjust treatments to optimise effectiveness. These reviews should entail validated saliva scale measures, in-depth questioning about contextual factors impacting saliva, and joint problem-solving to identify enablers to implement recommended strategies [18,20,23]. Our study highlighted successful adjustments to non-invasive measures (e.g., altered treatment dose/timing or identifying solutions to accommodate a change in physical function with disease progression). Similarly, direct questioning about side effects or inadequate effects of medical treatments identified when to escalate for prescription change. Thus, access to community clinicians knowledgeable in MND is essential to support saliva management [27,28].

In this study, the low completion rate of the saliva monitoring charts was not unexpected. Labra [29] reported ~12% completion rate of 3-day food diaries by plwMND, acknowledging the significant burden of multiple symptoms in this population. Exploring a less onerous electronic saliva diary/app, requiring minimum effort to complete, is warranted in future work.

### 4.6. Limitations and Future Directions

This pilot observational study provides only preliminary clinical outcome data based on patient-reported measures. The small geographically limited sample size, lack of a control group, and some incomplete data sets limit the strength and generalisability of findings. Sources of bias include some participants being known to the wider research team and satisfaction with resources being potentially amplified by the clinical intervention received at review appointments. However, a researcher independent of the MND clinic team (AC) collected data and used validated scales with standardised scoring to minimise risk of bias. Further, participants completed anonymous online surveys to minimise direct contact with researchers and limit potential positive response bias. Our public health study setting may have produced outcomes that would differ in a privately funded health context. Larger multi-centre studies, combining instrumental assessments (e.g., Flexible Endoscopic Evaluation of Swallowing (FEES)) with validated MND saliva scales, are required to objectively document saliva and secretions, to guide treatment, and measure outcomes. Future research should examine the treatment effects of non-invasive measures on different types and severities of saliva problems and examine perceptions of plwMND regarding the relative merits and burden of saliva treatments.

## 5. Conclusions

This study highlights the complexity of saliva and secretion problems faced by plwMND, necessitating structured and multifaceted management approaches. The pilot evaluation demonstrated that assessment of saliva characteristics and contextual factors helped identify treatment targets, and a hierarchical decision-making framework prompted consideration of non-invasive treatments as first-line management. Preliminary findings suggest frequent review of symptoms and treatment effects can inform adjustments, to continue non-invasive measures for some plwMND, or escalation to second-line treatments where necessary. High user satisfaction indicates that instructional resources can support plwMND to self-manage saliva symptoms, and sharing treatment information with community clinicians can optimise collaborative, person-centred care for plwMND.

## Figures and Tables

**Table 1:** Key characteristics of plwMND and their caregivers who participated in Phase 2 (*n* = 28).

Characteristic	No. of plwMND (%)
Sex	
Male	21 (67)
Female	7 (33)
MND onset site	
Bulbar	14 (50)
Limb	14 (50)
Months since diagnosis	
0–12	6 (21)
13–36	12 (43)
37+	10 (36)
Saliva/secretion characteristics (patient report and CSS-MND)	
Thin	17 (61)
Thick	10 (36)
Thin + thick	1 (4)
Saliva located in both the mouth and throat	25 (89)
Saliva only in the mouth	3 (11)

Note: CSS-MND = Clinical Saliva Scale-MND; plwMND = people living with MND.

**Table 2:** Frequency of treatments prescribed for saliva and saliva-related symptoms across time.

Treatments Prescribed	Time Point 0 Initial Session at MND Clinic	Time Point 1 Follow-Up	Time Point 2 Follow-Up	Time Point 3 Follow-Up	Total Treatments Prescribed (%)	
Behavioural strategies	39	29	20	13	101	(51)
Natural remedies	37	19	13	6	75	(38)
Pharmacological agents	10	1	6	3	20	(10)
Botulinum toxin injection	0	0	1	0	1	(0.5)
Radiation to salivary gland/s	1	0	0	0	1	(0.5)
Postnasal drip treatment	2	1	0	0	3	
Reflux treatment	1	2	0	1	4	
Thrush treatment	2	1	0	0	3	

**Table 3:** Multivariable model of changes in saliva symptoms (CSS-MND Total Score) (*n* = 27) *.

Variable		Β	Standard Error	z	*p*	95% Confidence Interval	
						Lower Bound	Upper Bound
Time point	1 (vs. 0)	−1.500	0.667	−2.25	0.025	−2.808	−0.192
	2 (vs. 0)	−2.568	0.694	−3.70	<0.0001	−3.928	−1.207
	3 (vs. 0)	−1.556	0.862	−1.80	0.070	−3.246	0.134
Saliva type	Thick (vs. thin)	1.719	2.025	0.85	0.396	−2.250	5.689
MND onset type	Limb (vs. bulbar)	−4.406	1.932	−2.28	0.023	−8.192	−0.619

Note. * One participant was excluded due to a mixed saliva type (unable to be dichotomised for analysis). CSS-MND = Clinical Saliva Scale-MND.

## Data Availability

Due to the nature of this study, participants did not agree for data to be shared publicly, and supporting data will therefore not be available.

## Supplementary Materials

The following supporting information can be downloaded at: https://www.mdpi.com/article/10.3390/healthcare13212813/s1, MND Saliva Rating Scale_additional questions; MND Saliva Management Resources Survey.
