# Supplementary material for: Co-Design and Non-Randomised Pilot Evaluation of Resources Developed to Optimise Saliva Management in People with Motor Neurone Disease
Source: Healthcare (Basel). 2025 Nov 5;13(21):2813. doi: 10.3390/healthcare13212813 (PMC12607367; doi:10.3390/healthcare13212813)
Supplement: Supplementary file 1 [file healthcare-13-02813-s001.zip › SUPP_MND Saliva Management Resources Survey.pdf]

Saliva Rating Scales – Person with MND

NAME: ________________________________ DATE: ___________________

Please circle the applicable rating.

How often does your mouth feel **dry**?

0	1	2	3	4
Never	Hardly ever	Occasionally	Fairly often	Very often

How much does changes in your saliva **affect your daily life**?

0	1	2	3	4
Never	Hardly ever	Occasionally	Fairly often	Very often

MND Saliva Resources Feedback Form – Person with MND

The following questions ask for your feedback on the saliva management plan and resources. We value your feedback. (Please circle response)

1. **The saliva management plan was easy to understand and follow.**

0<br>Strongly Disagree	1<br>Disagree	2<br>Neither agree or disagree	3<br>Agree	4<br>Strongly Agree

2. **The online saliva management resources on the RBWH website were easy to access.**

0<br>Strongly Disagree	1<br>Disagree	2<br>Neither agree or disagree	3<br>Agree	4<br>Strongly Agree

3. **The written saliva management resources (e.g. handouts) were easy to understand.**

0<br>Strongly Disagree	1<br>Disagree	2<br>Neither agree or disagree	3<br>Agree	4<br>Strongly Agree

4. **The videos on saliva management were easy to understand.**

0<br>Strongly Disagree	1<br>Disagree	2<br>Neither agree or disagree	3<br>Agree	4<br>Strongly Agree

5. **The online resources improved my understanding about saliva management.**

0<br>Strongly Disagree	1<br>Disagree	2<br>Neither agree or disagree	3<br>Agree	4<br>Strongly Agree

6. **The saliva management resources helped me to troubleshoot difficulties with my saliva.**

0<br>Strongly Disagree	1<br>Disagree	2<br>Neither agree or disagree	3<br>Agree	4<br>Strongly Agree

7. **I would recommend the online resources to other patients with MND**

0<br>Strongly Disagree	1<br>Disagree	2<br>Neither agree or disagree	3<br>Agree	4<br>Strongly Agree

Please provide any other feedback about the saliva plan and/or resources.

Thank you for your feedback.

MND Saliva Resources Feedback Form – Person with MND's caregiver

The following questions ask for your feedback on the saliva management plan and resources. We value your feedback. (Please circle response)

1. **The saliva plan provided to the person I care for, was easy to understand and follow.**

0	1	2	3	4
Strongly Disagree	Disagree	Neither agree or disagree	Agree	Strongly Agree

2. **The online saliva management resources on the RBWH website were easy to access.**

0	1	2	3	4
Strongly Disagree	Disagree	Neither agree or disagree	Agree	Strongly Agree

3. **The written saliva management resources (e.g. handouts) were easy to understand.**

0	1	2	3	4
Strongly Disagree	Disagree	Neither agree or disagree	Agree	Strongly Agree

4. **The videos on saliva management were easy to understand.**

0	1	2	3	4
Strongly Disagree	Disagree	Neither agree or disagree	Agree	Strongly Agree

5. **The online resources improved my understanding about saliva management.**

0	1	2	3	4
Strongly Disagree	Disagree	Neither agree or disagree	Agree	Strongly Agree

6. **The saliva management resources helped me to troubleshoot issues with the person I care for about difficulties with their saliva.**

0	1	2	3	4
Strongly Disagree	Disagree	Neither agree or disagree	Agree	Strongly Agree

7. **I would recommend the online resources to other carers of people with MND.**

0	1	2	3	4
Strongly Disagree	Disagree	Neither agree or disagree	Agree	Strongly Agree

Please provide any other feedback about the saliva plan and/or resources.

Thank you for your feedback.

MND Saliva Resources Feedback Form – Clinician

The following questions ask for your feedback on the saliva management plan and resources. We value your feedback. (Please circle response)

1. **The saliva plan provided to the person I care for, was easy to understand and follow.**

0<br>Strongly Disagree	1<br>Disagree	2<br>Neither agree or disagree	3<br>Agree	4<br>Strongly Agree

2. **The online saliva management resources on the RBWH website were easy to access.**

0<br>Strongly Disagree	1<br>Disagree	2<br>Neither agree or disagree	3<br>Agree	4<br>Strongly Agree

3. **The written saliva management resources (e.g. handouts) were easy to understand.**

0<br>Strongly Disagree	1<br>Disagree	2<br>Neither agree or disagree	3<br>Agree	4<br>Strongly Agree

4. **The videos on saliva management were easy to understand.**

0<br>Strongly Disagree	1<br>Disagree	2<br>Neither agree or disagree	3<br>Agree	4<br>Strongly Agree

5. **The online resources improved my understanding about saliva management.**

0<br>Strongly Disagree	1<br>Disagree	2<br>Neither agree or disagree	3<br>Agree	4<br>Strongly Agree

6. **The saliva management resources helped me to troubleshoot issues with my patients.**

0<br>Strongly Disagree	1<br>Disagree	2<br>Neither agree or disagree	3<br>Agree	4<br>Strongly Agree

7. **I would recommend the online resources to other clinicians working with people with MND.**

0<br>Strongly Disagree	1<br>Disagree	2<br>Neither agree or disagree	3<br>Agree	4<br>Strongly Agree

Are there any additional resources you would like developed? ________________________________

Please provide any other feedback about the saliva plan and/or resources.

____

____

Thank you for your feedback.
