# Supplementary material for: Co-Design and Non-Randomised Pilot Evaluation of Resources Developed to Optimise Saliva Management in People with Motor Neurone Disease
Source: Healthcare (Basel). 2025 Nov 5;13(21):2813. doi: 10.3390/healthcare13212813 (PMC12607367; doi:10.3390/healthcare13212813)
Supplement: Supplementary file 1 [file healthcare-13-02813-s001.zip › SUPP_MND Saliva Rating Scale_additional questions.pdf]

Saliva Rating Scales – Patient

NAME: ________________________________ DATE: ___________________

Please circle the applicable rating.

How often does your mouth feel **dry**?

0	1	2	3	4
Never	Hardly ever	Occasionally	Fairly often	Very often

How much does changes in your saliva **affect your daily life**?

0	1	2	3	4
Never	Hardly ever	Occasionally	Fairly often	Very often
